# Treatment and outcomes of tumor-induced osteomalacia associated with phosphaturic mesenchymal tumors: retrospective review of 12 patients

**DOI:** 10.1186/s12891-017-1756-1

**Published:** 2017-09-21

**Authors:** Qing-yao Zuo, Hong Wang, Wei Li, Xiao-hui Niu, Yan-hong Huang, Jia Chen, Yu-hua You, Bao-yue Liu, Ai-min Cui, Wei Deng

**Affiliations:** 1 Department of Endocrinology, Beijing Jishuitan Hospital, Xicheng District, Xinjiekoudongjie No. 31, Beijing, 100035 China; 2 Department of Orthopaedic Oncology, Beijing Jishuitan Hospital, Beijing, China; 3 Department of Rheumatology, Beijing Jishuitan Hospital, Beijing, China; 4 Department of Radiology, Beijing Jishuitan Hospital, Beijing, China; 5 Department of Pathology, Beijing Jishuitan Hospital, Beijing, China; 6 Department of General Surgery, Jishuitan Hospital, Beijing, China

**Keywords:** Oncogenic osteomalacia, Hypophosphatemia, Fractures, Spontaneous, Phosphaturic mesenchymal tumor, Fibroblast growth factor 23

## Abstract

**Background:**

Tumor-induced osteomalacia (TIO) is a rare paraneoplastic syndrome characterized by severe hypophosphatemia and osteomalacia. Nonspecific symptoms make the diagnosis elusive. In addition, locating the responsible tumor(s) is challenging. The aim of this study was to investigate the clinical management and outcomes of TIO.

**Methods:**

The clinical features, diagnostic procedures, treatment, and outcomes of 12 patients were reviewed retrospectively.

**Results:**

The cohort comprised six men and six women (mean age 45.5 ± 9.9 years, range 23–61 years). The mean duration of disease was 3.7 ± 2.6 years. All patients manifested progressive bone pain, muscle weakness, and/or difficulty walking. Serum phosphorus concentrations were low in all patients (mean 0.42 ± 0.12 mmol/L). Technetium-99m octreotide scintigraphy was performed in 11 patients and showed lesions in the right distal femur, left femoral head, and right tibial plateau, respectively, in three patients. Magnetic resonance imaging (MRI) was negative for lesions in one patient. Two patients underwent biopsies that showed negative histopathology. Two patients, at 2 years and 8 months, respectively, after having negative technetium-99m octreotide studies, underwent ^18^F–fluorodeoxyglucose positron emission tomography/computed tomography (CT), which revealed lesions in the sacrum and soft tissue of the left palm, respectively. One tumor was detected by CT and MRI. Overall, lesion sites were the head (two patients, 16.7%), thoracic and lumbar region (two, 16.7%), pelvis (three, 25%), lower limbs (four, 33.3%), and upper limbs (one, 8.3%). All patients underwent surgery, and histopathology showed phosphaturic mesenchymal tumors in each. Postoperatively, serum phosphorus concentrations normalized within 2–7 days in 11 patients. With follow-ups of 1–41 months, surgery was effective in 10 patients. One patient developed local recurrence and another had metastases.

**Conclusions:**

Locating tumors responsible for tumor-induced osteomalacia is often challenging. Although complete tumor resection confers a good prognosis in most patients, surveillance for recurrence and metastasis is necessary. Before surgery or when surgery is not indicated, oral phosphate can alleviate symptoms and metabolic imbalance.

**Electronic supplementary material:**

The online version of this article (10.1186/s12891-017-1756-1) contains supplementary material, which is available to authorized users.

## Background

Tumor-induced osteomalacia (TIO) is a rare paraneoplastic syndrome characterized by severe hypophosphatemia and osteomalacia related to abnormal tumor production of fibroblast growth factor 23 (FGF23) [1, 2]. Most of the tumors are phosphaturic mesenchymal tumors (PMTs), which are small and difficult to detect. Nonspecific symptoms, including fatigue, bone pain, and musculoskeletal weakness, make the diagnosis elusive, often leading to delays in treatment [3, 4]. Moreover, locating the responsible tumors can be challenging. In China, the detection rate for TIO is still low, and misdiagnoses or missed diagnoses occur frequently. We describe 12 patients with TIO and histologically similar tumors that presented in various anatomical locations and were difficult to diagnose, some of which recurred.

## Methods

### Patients

A retrospective review of 12 consecutive patients with TIO hospitalized from February 2013 to June 2016 in Beijing Jishuitan Hospital, Beijing, China was performed. All patients presented with symptoms of osteomalacia, including bone pain, fatigue, and fracture. All had typical biochemical features of hypophosphatemia and high alkaline phosphatase (ALP) concentrations. This study was approved by Beijing Jishuitan Hospital Institutional Review Board, and written informed consent was obtained from all participants in the study.

### Biochemical parameters

The patients’ medical records were retrospectively reviewed. Relevant biochemical variables, including serum ALP, calcium, phosphorus, 25-hydroxyvitamin D [25-(OH) D_3_] and parathyroid hormone (PTH), were extracted and recorded. In patient 11, the serum FGF23 C-terminal fragment concentration was measured using an enzyme-linked immunosorbent assay (Immtopics, San Clemente, CA, USA). Spot urine samples had been collected to assess tubular reabsorption of phosphorus:

1 − (urine phosphorus concentration × serum creatinine concentration) ÷ (plasma phosphorus concentration × urine creatinine concentration).

The tubular maximum reabsorption of the phosphate/glomerular filtration rate (TMP/GFR) ratio was calculated using the Walton–Bijvoet nomogram [5].

After performing a thorough physical examination, various methods were used to detect the causative tumors, including somatostatin receptor-based functional imaging [e.g., technetium-99m octreotide (^99m^Tc-OCT) scintigraphy] and ^18^F–fluorodeoxyglucose positron emission tomography/computed tomography (^18^F–FDG-PET/CT). Anatomical imaging methods [e.g., magnetic resonance imaging (MRI), CT, ultrasonography] were carried out to locate the responsible tumors. All patients with identified bone and/or soft tissue tumors were treated surgically.

Successful treatment was defined as alleviation of symptoms and normalization of serum phosphorus concentrations. Postoperative follow-up included symptom assessment, serum phosphorus levels, and assessment for tumor relapse.

### Statistical analysis

Data were analyzed using statistical software SPSS version 16.0 (SPSS, Chicago, IL, USA). Normally distributed variables are expressed as means ± standard deviation (SD) and values with skew distribution as medians (range). Paired *t*-tests were performed to compare changes in serum phosphorus concentrations before and after phosphate supplementation. Differences were considered significant when *p* < 0.05.

## Results

### Baseline characteristics

The cohort comprised six women and six men (mean age 45.5 ± 9.9 years, range 23–61 years at the time of diagnosis). The mean duration of bone pain before diagnosis was 3.7 ± 2.6 years (range 0.5–10 years) (Table 1). Nine of the patients reported shortened stature of 4.3 ± 2.7 cm (range 2–12 cm) after the onset of bone pain.

**Table 1:** Clinical features and imaging examinations of 12 patients with TIO

Case	Gender	Duration (y)	Calcium (mmol/L)	25-(OH)D_3_ (ng/ml)	PTH (pg/ml)	Phosphorous (mmol/L)	ALP (IU/L)	24hUP (mmol)	RP (d)	PE	Localization methods		Further localization		Tumor location	Pathology
											OCT	PET/CT	MRI/CT	US		
1	F	4	2.14	21.92	74.1	0.53	94	18.33	7	N	P	P	P	–	lumbar vertebrae 1	PMT
1^a^	F	0.5	1.98	13.13	59.5	0.45	89	18.32	3	N	P	P	P	–	lumbar vertebrae 1	PMT
2	F	4	2.01	14.09	69.6	0.33	95	35.82	2	N	N	P	P	–	intra sacral canal	PMT
3	F	0.5	2.27	11.27	67.1	0.69	176	16.97	3	N	–	–	P	–	right ilium	PMT
4	F	5	2.14	18.77	54.3	0.38	264	4.98	3	N	P	–	P	P	soft tissue of left thigh	PMT
5	M	3	2.12	16.33	71	0.24	96	18.76	3	N	P	–	P	–	soft tissue of left nasal cavity	PMT
6	M	2	2.18	16.72	41.9	0.41	237	37.26	3	N	P	–	P	P	soft tissue of left thigh	PMT
7	F	3	2.05	4.22	57	0.44	98	20.08	3	N	N	P	P	–	left maxillary bone	PMT
8	M	4	2.02	7.38	56.5	0.51	236	33.64	3	N	N	P	P	–	right lateral proximal femur	PMT
9	M	7	2.26	14.78	70.2	0.44	479	9.62	3	P	N	P	–	P	left palm soft tissue	PMT
10	F	2	2.42	6.66	56.3	0.36	162	10.01	2	P	P	–	P	P	mons veneris area soft tissue	PMT
11	M	10	2.65	23.06	172	0.28	87	8.50	not recover-ed	N	P	P	P	P	left popliteal fossa, left proximal tibiofibula soft tissue, right adductor magnus	PMT
12	M	3	2.39	14.18	74.9	0.45	233	43.42	3	N	N	P	P	–	the 5th rib on the left side	PMT
Summary	6/6	3.7 ± 2.6	2.20 ± 0.19	14.04 ± 5.64	71.1 ± 31.8	0.42 ± 0.12	180.5 ± 112.3	21.21 ± 12.40	3 (2, 7)	2	7/12	8/8	12	5	2 in head, 2 in thoracic and lumber region, 3 in pelvis, 4 in lower limbs, 1 in upper limbs	

^a^: Patient 1 recurrence; *24-h UP* 24 h urine phosphorus, *RP* recovery time of serum phosphorus after surgery, *OCT* ^99m^Tc-octreotide, *PE* physical examination, *US* ultrasonography;

*P* positive, *N* negative; −, not performed

Values with normal distribution are expressed as the mean ± SD; values with skew distribution are expressed as median (range)

Symptoms included proximal muscle weakness (100%), general debility (100%), and progressive bone pain (100%) involving the dorsal mid-thorax, lower anterior chest wall, bilateral flanks, and sacroiliac joint and ankle bone areas in the absence of antecedent trauma. Fifty percent of patients had at least one pathological fracture caused by osteomalacia (including vertebral body, rib, and pelvic fractures). Patients 1, 2, 4, 5, 8, 9, 10, and 12 had difficulty walking at the time of referral to our hospital.

**Fig. 1:**
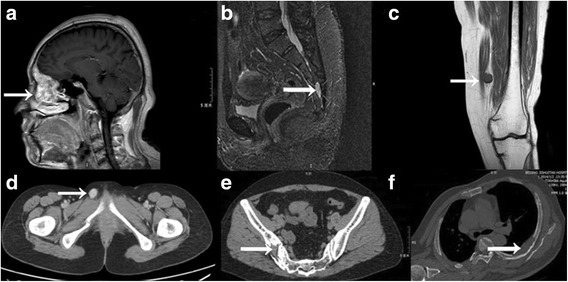
Magnetic resonance imaging (MRI) and computed tomography (CT) of causative tumors. (**a**) Patient 5. Cranial MRI showed a soft mass (arrow) in the left nasal cavity and ethmoid, frontal, and maxillary sinuses. (**b**) Patient 2. MRI showed a tumor (arrow) in the sacral canal. (**c**) Patient 4. MRI showed a tumor (arrow) in the soft tissue of the left thigh. (**d**) Patient 10. CT showed a neoplasm (arrow) in the mons pubis. (**e**) Patient 3. CT showed a mass (arrow) in the right ilium. (**f**) Patient 12. CT showed a tumor (arrow) in the left fifth rib

**Fig. 2:**
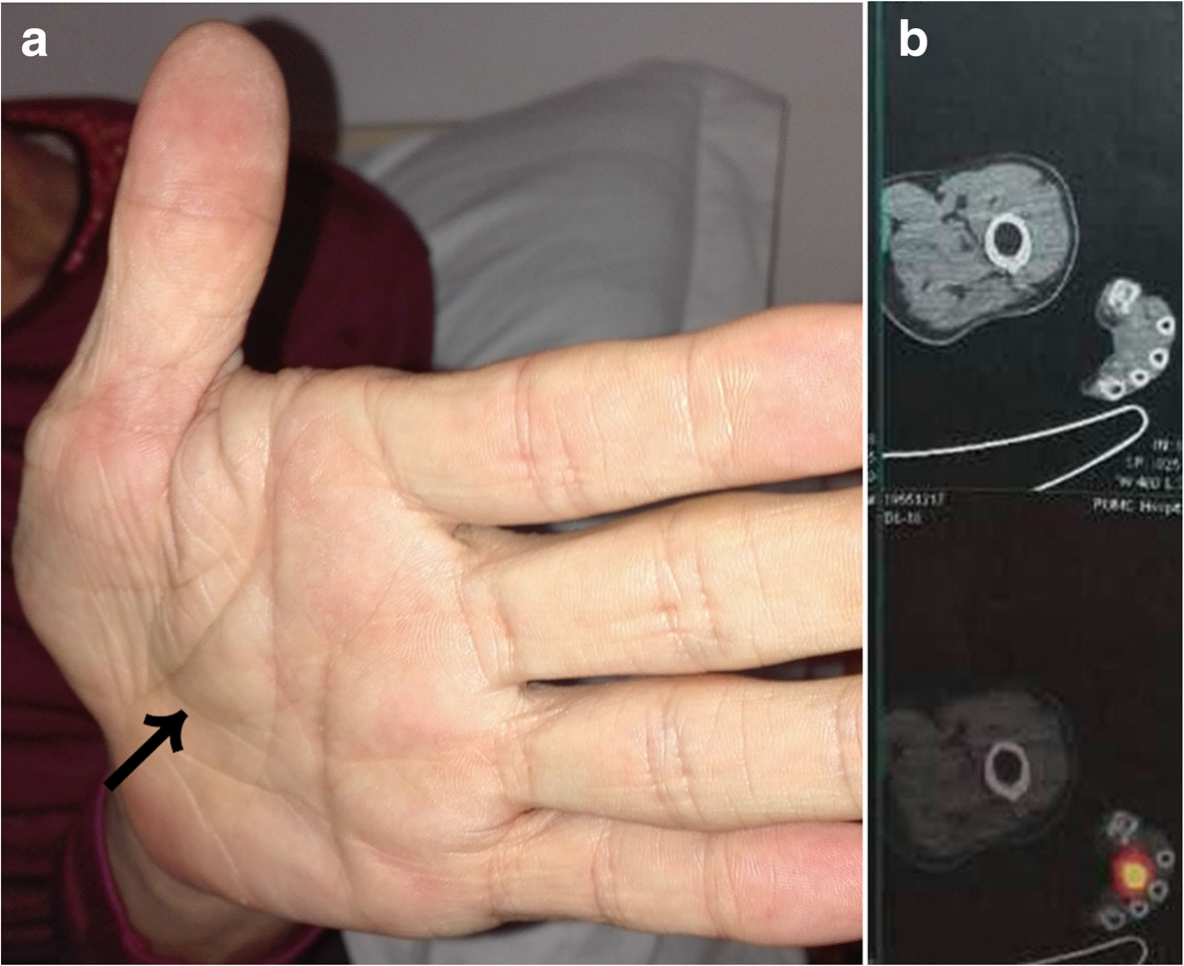
Patient 9. (**a**) A tumor was visible and palpable in the soft tissue of the left palm (arrow). (**b**) ^18^F–FDG PET/CT showed high ^18^F–FDG uptake in the tumor

Prior to referral, 11 (91.7%) patients had been misdiagnosed as having rheumatoid arthritis, ankylosing spondylitis, spondylolisthesis, osteoporosis, or sacroiliitis. Patient 12 had been diagnosed as having depression. Patient 5 had presented to the primary hospital with progressive bone pain in the low back and hip, difficulty walking, muscle weakness, and epistaxis. He underwent whole-body MRI, head CT, arthroscopy, bone marrow biopsy, and PET/CT, resulting in provisional diagnoses of maxillary sinusitis and myeloma (Fig. 1). Patient 10 had had multiple fractures for 6 months when she presented to our hospital. Patient 10 was found on physical examination to have a mass in the mons pubis (Fig. 1). Physical examination also confirmed a mass that had been growing slowly in the left palm of patient 9 for 7 years (Fig. 2a).

### Laboratory findings

Relevant laboratory findings were as follows. Serum phosphorus concentrations were low in all 12 patients (mean 0.42 ± 0.12 mmol/L; normal range 0.84–1.65 mmol/L), whereas serum calcium concentrations were normal in five patients and slightly decreased in seven (mean 2.20 ± 0.19 mmol/L; normal range 2.20–2.65 mmol/L); serum ALP 180.5 ± 112.3 U/L (normal range 40–150 U/L); serum PTH 71.1 ± 31.8 pg/mL (normal range 15.0–65.0 pg/mL); 25-(OH)D_3_ 14.04 ± 5.64 ng/mL (normal range 20.00–40.00 ng/mL); 24-h urine phosphorus 21.21 ± 12.40 mmol (normal range 9.70–43.40 mmol) (Table 1); and TMP/GFR 0.31 ± 0.12 mmol/L (*n* = 9, normal range 0.80–1.35 mmol/L). For patient 11, the serum FGF23 concentration was increased both before and after the last surgery: 2007.85 and 1795.35 RU/ml, respectively (normal range ≤ 180 RU/ml).

### Identification of locations of tumors

CT revealed a tumor in one patient (patient 3), who had presented to the primary hospital with hematuria. MRI was used to further characterize a lesion on the iliac side of the right hip joint.

**Fig. 3:**
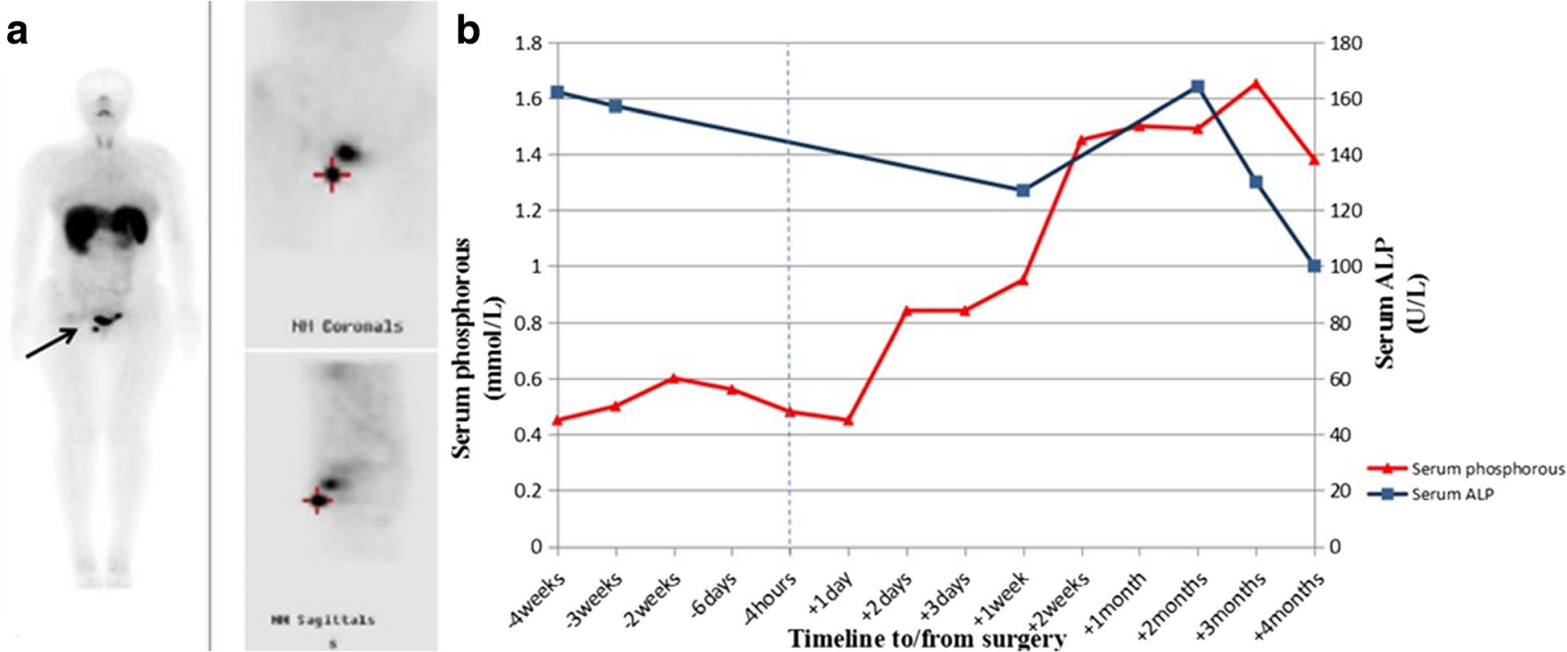
Patient 10. (**a**) ^99m^Tc-OCT showed strong expression of somatostatin receptors in the patient’s right mons pubis (arrow). (**b**) Recovery of serum phosphorus and alkaline phosphatase (ALP) in patient 10. The time of surgery is represented by the dashed line. Normal range of serum phosphorus was 0.84–1.65 mmol/L. Normal range of serum ALP was 40–150 U/L.

^99m^Tc-OCT scans were performed in 11 patients (patient 1 had two examinations, the second because of recurrence) (Table 1), which revealed tumors in 9 of them (Fig. 3a). The scans were negative in the remaining two patients (nos. 2 and 9). Tumors were identified by ^18^F–FDG PET/CT in the sacrum and soft tissue of the left palm (Fig. 2b) of these patients after 2 years and 8 months, respectively.

Lesions were located by ^99m^Tc-OCT scintigraphy in the right distal femur, left femoral head, and right tibial plateau in patients 7, 8, and 12, respectively. MRI had failed to identify a tumor in patient 8, and biopsy specimens obtained from patients 7 and 12 had been negative for tumor cells. Subsequently, PET/CT identified the tumors in different locations. Other forms of anatomical imaging (e.g., MRI, CT, ultrasonography) were used to verify their locations (Fig. 1). There were no obvious clinical differences between patients whose tumors were identified by ^99m^Tc-OCT scintigraphy versus ^18^F–FDG PET. In patient 11, ^99m^Tc-OCT showed multiple tumors in the soft tissue of the right inner thigh and left proximal crus.

The final sites of the lesions were the head (two patients, 16.7%), thoracic and lumbar region (two, 16.7%), pelvis (three, 25%), lower limbs (four, 33.3%), and upper limbs (one, 8.3%) (Table 1). In this cohort, ^99m^Tc-OCT scintigraphy detected tumors accurately in 7 of 12 patients (58.3%).

### Treatment

Before surgery, phosphate supplements, calcium carbonate, and active vitamin D (calcitriol or α-calcitriol) were administered to all patients except nos. 3 and 12. Although all patients remained hypophosphatemic, serum phosphorus concentrations were significantly higher in those who were given the supplements (0.38 ± 0.08 vs. 0.62 ± 0.07 mmol/L, *p* < 0.001), and their symptoms progressively diminished. Patients 2 and 9 were treated with a regimen of phosphate (1.2 g/day) and calcitriol (0.5 μg/day) for 2 years and 8 months, respectively. They each gradually became capable of walking on crutches instead of being bedridden.

All 12 patients underwent tumor resection, after which the serum phosphorus concentrations normalized in 11 of them, the exception being patient 11. The median time to normalization of the phosphorus concentration was 3 days (range 2–7 days) (Table 1, Fig. 3b). The TMP/GFR increased to within the normal range in patient 1 (1.16 mmol/L, after secondary surgery) and patient 10 (1.10 mmol/L).

### Histopathology

**Fig. 4:**
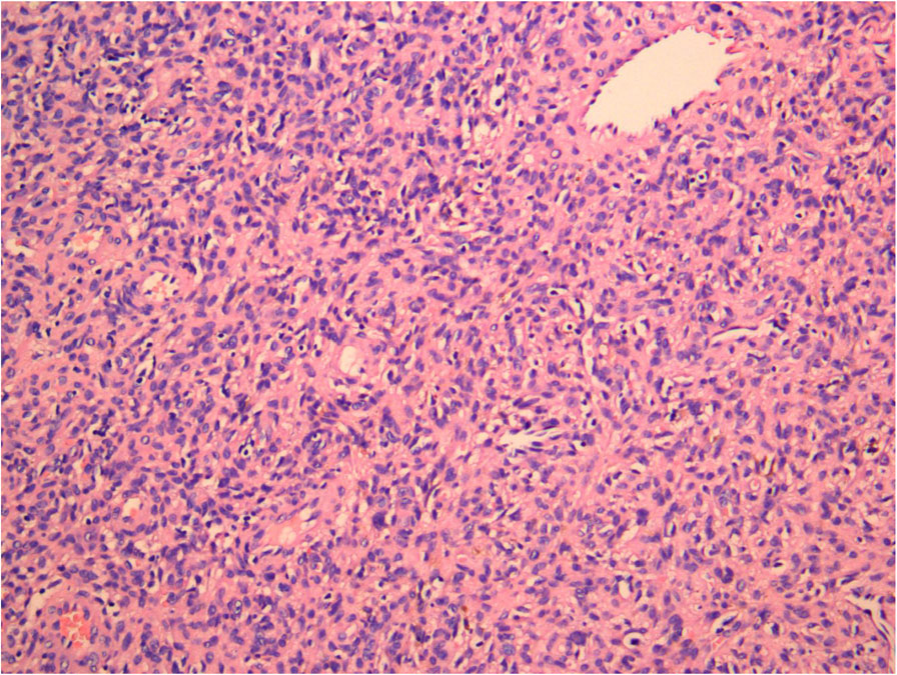
Patient 11. Histological features of a phosphaturic mesenchymal tumor (PMT). Spindle mesenchymal cells are mildly atypical, with low mitotic activity and irregular vessels (H&E, original magnification ×20)

Twelve patients were confirmed to have TIO by pathology examination of the resected specimens. All 12 tumors were PMTs. Their dominating components were spindle/stellate mesenchymal cells with mild atypia (Fig. 4) that produced unusual grungy or flocculent calcification (Additional file 1: Figure S1a). These tumors often had a distinctive myxoid/myxochondroid matrix (Additional file 1: Figure S1b) with highly irregular blood vessels embedded in it. In some instances, the spindle cells surrounded “staghorn” vessels in a pericytoma-like pattern (Additional file 1: Figure S1c). There was no necrosis.

### Follow-up

The mean follow-up was 22.4 ± 13.6 months (range 1–41 months). Bone pain and muscle weakness diminished within 3–6 months postoperatively in all patients except no. 11. Patient 1 had a local recurrence 34 months after the first surgery.

### Tumor relapse

Patient 1 underwent tumor resection twice. After the first surgery, her serum phosphorus level normalized within 1 week. However, 34 months later, she again developed lumbar and thigh pain and was found to have a low serum phosphorus level with normal calcium concentrations, high serum ALP, low 25-(OH)D_3_, and urinary phosphate loss. ^99m^Tc-OCT and PET/CT indicated local recurrence in the original location. She again underwent resection. Her serum phosphorus increased to 1.45 mmol/L within 3 days of the surgery. The pathological features of PMT were found, suggesting TIO recurrence.

Patient 11, a 40-year-old man, underwent five surgeries over 10 years. He initially presented with generalized muscle pain at the age of 30 years. Eight years ago, he developed additional pain in the hip and thigh muscles. Laboratory testing showed a low serum phosphorus level (0.13 mmol/L). Plain radiography revealed pseudofractures. ^99m^Tc-OCT showed a mass in the fifth metatarsus of the left foot, for which he underwent surgery. Then, 6 years ago, ^99m^Tc-OCT demonstrated a neoplasm in the soft tissue behind the fifth and fourth metatarsals of the left foot and repeat surgery was performed. Two years later, a tumor was found in soft tissue adjacent to the proximal right femur, for which a third excision was performed, alleviating his symptoms. The tumor was pathologically identified as PMT. Two years ago, another tumor was found in soft tissue adjacent to the left proximal tibia and fibula. After this fourth excision, however, his symptoms failed to lessen. He underwent further evaluation, during which ^99m^Tc-OCT, MRI, and ultrasonography revealed lesions in soft tissue adjacent to the left proximal tibia and fibula and the left popliteal fossa as well as multiple neoplasms in the right adductor magnus. Despite resection of one of the tumors in the adductor magnus, his symptoms and hypophosphatemia persisted.

## Discussion

TIO, also known as oncogenic osteomalacia, is an uncommon cause of osteomalacia. This rare paraneoplastic syndrome is characterized by acquired hypophosphatemic osteomalacia caused by a deficit in renal tubular phosphate reabsorption in which FGF23 seems to be implicated [6]. Since the first report by Prader et al. in 1959 [7], TIO has been increasingly reported worldwide [4, 8]. This condition is usually associated with PMT of mixed connective tissue located in bone or soft tissue. The main biochemical manifestations of this disorder include hypophosphatemia, inappropriately low or normal tubular resorption of phosphate, low serum calcitriol concentrations, high serum alkaline phosphatase, and high or normal serum FGF-23 concentrations [9] .

The clinical manifestations of TIO include fatigue, muscle weakness, bone pain, and spontaneous fractures in the absence of a family history of renal phosphate wasting and/or bone disorders. Disease progress can be slow and insidious, with the correct diagnosis often delayed for years. Among our 12 patients, 11 were initially misdiagnosed as having a rheumatological, endocrine, or neurological disorder because the manifestations were so vague. Without intervention, patients become progressively debilitated, experience a markedly decreased quality of life, and are at risk of life-threatening complications [10].

In the present study, all 12 patients initially had adult-onset osteomalacia (average age 45.5 ± 9.9 years). The sex distribution was balanced (six men, six women). The patients’ presenting symptoms were often common and nonspecific. Progressive bone pain and muscle weakness can be incorrectly attributed to many diseases, including rheumatoid arthritis, ankylosing spondylitis, spondylolisthesis, and osteoporosis. Patient 5 was referred to the primary hospital because of progressive bone pain, difficulty walking, muscle weakness, and epistaxis. Findings on whole-body MRI, head CT, arthroscopy, bone marrow biopsy, and PET/CT resulted in the provisional diagnoses of sinusitis and myeloma. Hypophosphatemia and renal wasting of phosphorus, which can be identified by calculating the TMP/GFR, are often the first findings to suggest the possibility of TIO. Hence, we suggest that, once TIO is suspected, conventional biochemical tests, including serum phosphorus, calcium, and ALP levels, should be performed. A high serum FGF23 concentration is suggestive of TIO.

One of the most challenging aspects of managing patients with TIO is identifying the causative tumor, which is often delayed because of the occult nature of TIO [11]. The tumors are characteristically small (usually 1–4 cm) and may occur in unusual sites. Frequent locations are the lower limbs, head, upper limbs, thorax, abdomen, and pelvis. Patients 2’s tumor was in the sacral canal, which is exceptionally rare.

Although PMT is regarded as benign, the lengthy interval from the onset of symptoms to diagnosis and its potential for recurrence may be associated with considerable morbidity [12]. When oncogenic osteomalacia is suspected, it is important to search carefully for tumor foci with ^99m^Tc-OCT scintigraphy because these tumors often remain undetected by conventional tests [13]. ^99m^Tc-OCT scintigraphy is used mainly to detect areas of active osteomalacia rather than to locate the tumor(s) [14, 15]. In our cohort, ^99m^Tc-OCT was used in attempts to diagnose and locate the tumors in 11 of 12 patients. In two patients, the tumors were uptake-negative. They were ultimately identified by ^18^F–FDG PET/CT, which was the primary diagnostic imaging modality in eight patients. In our study, the accuracy rate of ^99m^Tc-OCT was 58.3%, which is similar to that reported by Yu et al. (60%) [8]. Possible explanations for the initial failure to identify tumors in five patients include the tumors’ elusive nature and the fact that different tumors have different degrees of expression of somatostatin receptor subtypes and different affinities for somatostatin analogs. Because lymphocytes can express octreotide receptors, nonspecific uptake attributable to inflammatory tissues, fractures, or other tumors may cause false-positive results. Negative or false-positive ^99m^Tc-OCT scans necessitate further investigation using PET/CT [16, 17]. Jadhav et al. reported that ^68^Ga-DOTA-TATE PET/CT may be more sensitive than ^18^F–FDG PET/CT because of its high affinity for somatostatin receptors [18]. ^68^Ga-DOTA-TATE studies were not performed on our patients because we lacked the required equipment.

Despite the superiority of the functional imaging already mentioned, our findings emphasize the diagnostic value of thorough physical examinations, which helped us detect the causative tumors in the two patients who had palpable and visible lesions. The oral and nasal cavities and the extremities should be given special attention. In fact, 3 of the 12 tumors (25%) (patients 5, 7, 9) identified in our study were in one of these locations.

One of our patients presented to the primary hospital with hematuria in the absence of bone pain or difficulty walking. This patient had hypophosphatemia, and the pathological diagnosis was PMT. Radiological examination, however, failed to reveal the tumor on the iliac side of the right hip joint that was later detected by MRI. This combination of findings suggests that prompt diagnosis and treatment of TIO could minimize bone damage.

Definitive treatment of TIO is surgical resection [19, 20]. All 12 patients underwent surgery, which led to resolution of symptoms in all patients and normalization of laboratory values in 11 of them. A key point is that total resection of the tumor is essential. The local recurrence in patient 1 indicated that the causative tumor had not been completely removed at the first operation. In our patients, serum phosphorus concentrations increased to normal within 2–7 days of the surgery and resolution of generalized bone and muscle pain with 3–6 months, confirming the appropriateness of the surgery.

Patients with hypophosphatemic osteomalacia of unidentified cause or whose tumors have not yet been detected should be treated with phosphorus supplementation and calcitriol because the causative tumors may remain undetectable for a long time. Medical therapy, however, is not effective in the long term. Therefore, such patients should be monitored for potential complications (i.e., hyperparathyroidism, hypercalcemia, kidney stone formation) [21].

Pathology examination of our patients’ resected tumors indicated that they were PMTs and benign, consistent with previously reports [21, 22]. One patient (no. 11), however, developed a mass in the first lumbar vertebra 3 years after the initial surgery. Serum phosphorus concentrations increased to normal after a second resection, and his bone pain resolved within 2 months. Sun et al. [19] reported that complete resection of soft tissue tumors is generally straightforward because they usually have clear boundaries and are well encapsulated. Patient 11 had numerous soft tissue tumors in different locations over a 10-year period. After the third operation, his symptoms and hypophosphatemia resolved. The fourth operation was ineffective, however, as only one of multiple soft tissue tumors situated in the right inner thigh, left proximal crus, and popliteal space was resected. After this procedure, his serum phosphorus concentration did not return to normal, and the FGF23 concentration remained dramatically high. This clinical course suggests malignancy.

TIO remained undiagnosed for several years in our patients. Careful metabolic evaluation and extensive investigations eventually led to the correct diagnoses and cure of the TIO by excising the offending tumors. We emphasize considering TIO early in the differential diagnosis of unexplained persistent bone pain, apparent joint pain, muscle weakness, and spontaneous fractures. We recommend the following investigations on initial presentation of patients with suspected TIO: serum phosphorus, 24-h urinary phosphorus, serum calcium, vitamin D, and FGF23 levels and either ^99m^Tc-OCT scintigraphy or PET/CT.

Definitive treatment is early, complete resection. The prognosis of PMT is generally good, although this series illustrates the pitfalls of delayed definitive resection. The FGF-23 level is useful for the diagnosis and may also prove to be beneficial for long-term monitoring of these patients.

The prognosis of patients with osteogenic osteomalacia is excellent in most cases, reflecting the generally benign nature of the disease. Nonetheless, two of our patients had recurrent or metastatic disease. Hence, vigilant postoperative follow-up is necessary.

The present series does have several limitations. The lack of FGF23 and 1,25-dihydroxyvitamin D assessments hindered the early diagnosis of TIO. In our future studies, FGF23 will be assessed in all patients suspected of having TIO.

## Conclusions

TIO is uncommon, and identification of the responsible tumor(s) is often challenging. Although complete tumor resection achieves a good prognosis in most cases, these patients should be monitored for recurrence and metastasis. Preoperatively or if surgery cannot be performed, oral medication with phosphate can alleviate symptoms and the metabolic imbalance.

## Additional file

Additional file 1: Figure S1. Histological features of a PMT in patient 10. (a) Grungy or flocculent calcification produced by spindle cells. (b) Myxoid/myxochondroid matrix. (c) Spindle cells surround the “staghorn” vessels in a pericytoma-like pattern (H&E, original magnification ×20). (BMP 3072 kb)

## Abbreviations

^18^F–FDG: ^18^F–fluorodeoxyglucose
24hUP: 24-h Urine phosphorus
25-(OH) D_3_: 25-Hydroxyvitamin D
^99m^Tc-OCT: Technetium-99m octreotide
ALP: Alkaline phosphatase
CT: Computed tomography
FGF23: Fibroblast growth factor 23
MRI: Magnetic resonance imaging
PE: Physical examination
PET: Positron emission tomography
PMT: Phosphaturic mesenchymal tumor
PTH: Parathyroid hormone
RP: Recovery time of serum phosphorus after surgery
TIO: Tumor-induced osteomalacia
TMP/GFR: Tubular maximum reabsorption of phosphate/glomerular filtration rate
