# Supplementary figures and images for: Treatment and outcomes of tumor-induced osteomalacia associated with phosphaturic mesenchymal tumors: retrospective review of 12 patients
Source: BMC Musculoskelet Disord. 2017 Sep 21;18:403. doi: 10.1186/s12891-017-1756-1 (PMC5609032; doi:10.1186/s12891-017-1756-1)

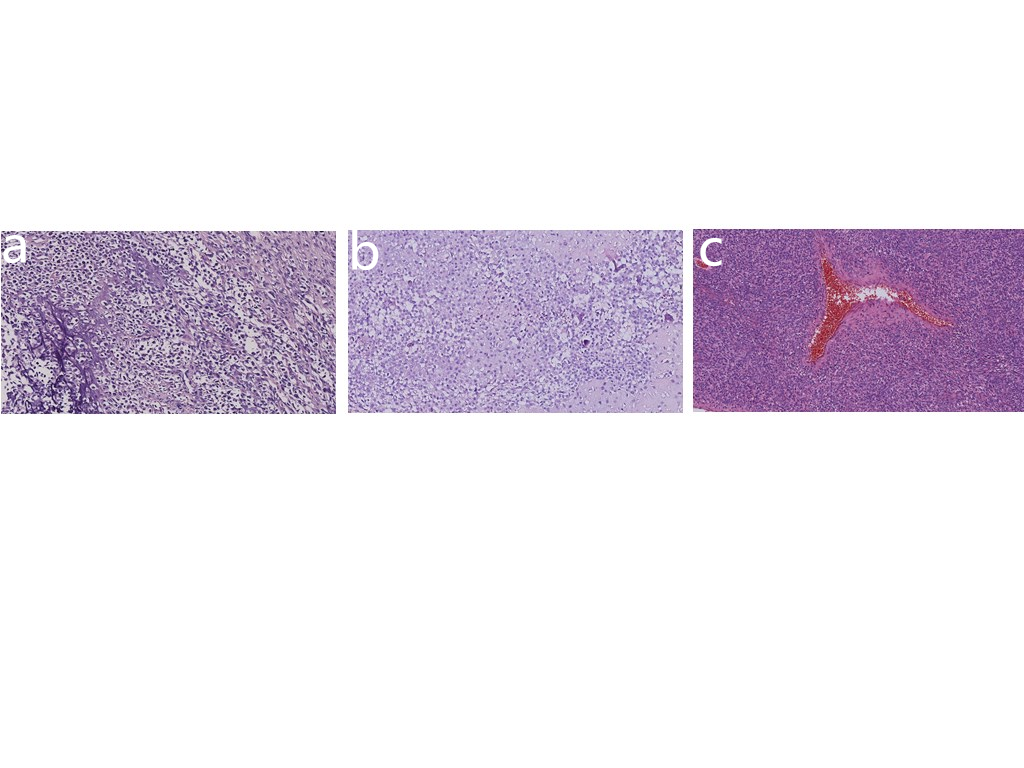

Supplement: Additional file 1: Figure S1. — Histological features of a PMT in patient 10. (a) Grungy or flocculent calcification produced by spindle cells. (b) Myxoid/myxochondroid matrix. (c) Spindle cells surround the “staghorn” vessels in a pericytoma-like pattern (H&E, original magnification ×20). (BMP 3072 kb) [file 12891_2017_1756_MOESM1_ESM.bmp]
